# Circulating tumor DNA: a promising biomarker in the liquid biopsy of cancer

**DOI:** 10.18632/oncotarget.9453

**Published:** 2016-05-19

**Authors:** Feifei Cheng, Li Su, Cheng Qian

**Affiliations:** ^1^ Institute of Pathology and Southwest Cancer Center, Southwest Hospital, Third Military Medical University, Chongqing, China; ^2^ School of Life Science, Zhejiang Sci-Tech University, Hangzhou, China

**Keywords:** liquid biopsy, circulating tumor DNA, biology, biomarker, targeted therapies

## Abstract

Tissue biopsy is the standard diagnostic procedure for cancers and also provides a material for genotyping, which can assist in the targeted therapies of cancers. However, tissue biopsy-based cancer diagnostic procedures have limitations in their assessment of cancer development, prognosis and genotyping, due to tumor heterogeneity and evolution. Circulating tumor DNA (ctDNA) is single- or double-stranded DNA released by the tumor cells into the blood and it thus harbors the mutations of the original tumor. In recent years, liquid biopsy based on ctDNA analysis has shed a new light on the molecular diagnosis and monitoring of cancer. Studies found that the screening of genetic mutations using ctDNA is highly sensitive and specific, suggesting that ctDNA analysis may significantly improve current systems of tumor diagnosis, even facilitating early-stage detection. Moreover, ctDNA analysis is capable of accurately determining the tumor progression, prognosis and assisting in targeted therapy. Therefore, using ctDNA as a liquid biopsy may herald a revolution for tumor management. Herein, we review the biology of ctDNA, its detection methods and potential applications in tumor diagnosis, treatment and prognosis.

## INTRODUCTION

Current tumor diagnosis depends on a variety of pathological examinations, among which, tissue biopsy is considered to be the gold standard. However, tissue biopsy-based tumor diagnosis has many limitations. For instance, the detection of early-stage tumor or residual lesions is unsatisfactory, and its application in the evaluation of treatment efficacy and prognosis is also limited [1–2]. With the continuous emerging of tumor-specific molecules, the interest in molecular diagnosis of tumors is rapidly increasing. Studies have found that tumor-relevant protein molecules and miRNAs as well as circulating tumor cells (CTC) are all suitable tumor biomarkers in the liquid biopsy of cancer [3–9]. However, sensitivity and specificity of these biomarkers remain suboptimal [10, 11], which impede their widespread application to clinical practice. Therefore, the identification of new, highly sensitive and specific tumor biomarkers is particularly important.

Circulating tumor DNA (ctDNA) is released by the tumor cells into the blood and thus harbors the mutations of the original tumor [12]. In the past decade, groundbreaking studies on ctDNA have been carried out, facilitated by advances in the cancer genome project (CGP) and new applications of next generation sequencing (NGS) technology. In addition to being noninvasive, researchers have found that the screening of genetic lesions using ctDNA is highly sensitive and specific [2, 10], suggesting that the use of ctDNA as a liquid biopsy may significantly improve current systems of tumor diagnosis, even facilitating early-stage detection. Moreover, ctDNA analysis is able to accurately determine the tumor progression, prognosis and assist in targeted therapy [13–17]. With the achievements of CGP and wide application of NGS, there is a growing expectation that liquid biopsy based on ctDNA analysis heralds a revolution for cancer diagnosis, prognosis and treatment.

## THE RESEARCH HISTORY OF ctDNA

In 1977, researchers made the novel observation that cancer patients carried cell-free DNA in their peripheral blood [18]. Initial progress on further characterization of cell-free DNA was frustratingly slow due to the technological limitations of the pre-genome era. It would be another 17 years until researchers proved unequivocally that this species of nucleic acid was derived from tumor tissues by virtue of the presence of characteristic cancer mutations [12, 19]. Indeed, significant progress was not made until recent 10 years with the advent of NGS technology in combination with the early findings of CGP, which significantly improved the sensitivity and specificity of ctDNA detection [13, 20]. Subsequently, research in this field has entered a “golden age” in which the huge potential of ctDNA investigations in tumor diagnosis and treatment is becoming ever clear [2, 21–24].

## THE BIOLOGY OF ctDNA

### The biological characteristics of ctDNA

ctDNA is single- or double-stranded DNA, and exists in plasma or serum. Early studies showed that ctDNA possessed many cancer-associated molecular characteristics, such as single-nucleotide mutations [25–29], methylation changes [30–33] and cancer-derived viral sequences [34–36], and therefore were considered to be derived from tumor tissue. These findings were significant for the development of future ctDNA detection technology [37–40]. In recent years, researchers have further demonstrated the potent applications of ctDNA in clinical practice. However, many biological characteristics of ctDNA remain unclear. For instance, the size of ctDNA fragments is still undetermined: some believe that it is longer than corresponding non-tumor cell-free DNA (cfDNA) [41, 42], and others believe the opposite [27, 43]. Such controversy might be explained by differences in the detection methods and sample sources used by different groups. In a recent study, Jiang et al. found that the plasma of liver cancer patients harbored both extremely long and short DNA molecules, and the short fragments tended to contain the tumor-relevant copy number aberrations [44]. Madhavan et al. reported a similar phenomenon in breast cancer patients [45]. These studies suggest that ctDNA is shorter than non-cancer cell-free DNA, and take an important step in solving the controversy. Additionally, whether ctDNA exists as complex is also a current study focus [46].

### The mechanism of ctDNA entry into the bloodstream

**Figure 1:**
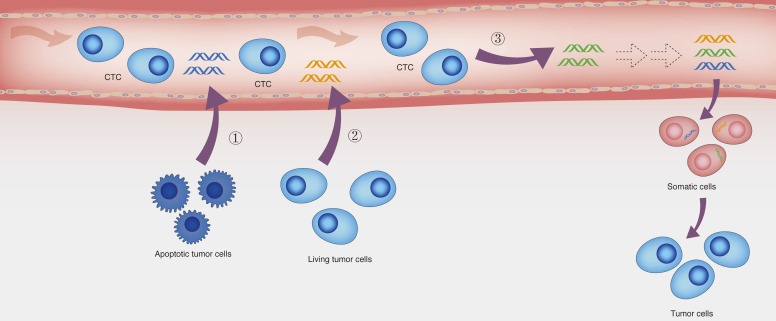
The potential origins of ctDNA and the hypothesis of genometastasis Three potential origins of ctDNA: (1) apoptotic tumor cells, (2) Living tumor cells, and (3) circulating tumor cells. Additionally, according to the hypothesis of genometastasis, ctDNA may transform normal cells to tumor cells, resulting in distant metastasis.

Though the existence of ctDNA is widely accepted, the mechanisms by which tumor DNA enters the bloodstream remain unclear. It has been suggested that there are three potential origins of ctDNA (Figure 1): 1) apoptotic or necrotic tumor cells, 2) living tumor cells, and 3) circulating tumor cells [46–49]. Actually, it is very likely that there are multiple origins for ctDNA rather than just one.

Several previous studies have reported that fragment sizes of cfDNA are around 166 bp, similar to those released by typical apoptotic cells [46–47]. Consistently, cfDNA display a ladder-like distribution after electrophoresis [48]. It has been widely accepted that the deregulation of proteolytic activities involved in apoptosis can lead to the release of DNA or nucleosomes into the blood circulation [50, 51]. Indeed, Roth C et al. found, the observed changes in apoptosis-related deregulation of proteolytic activities are along with the elevated level of DNA in blood [50]. Intriguingly, circulating nucleosome was significantly associated with DNA concentrations in the blood of patients and healthy subjects [50], which was supported by the previous observations of Holdenrieder S, et al. [52]. Based on the above findings, ctDNA may be released by apoptotic cells in the form of nucleosomes. Although most of the liberated nucleosomes are engulfed and digested by macrophages, this elimination system can be overloaded or impaired in case of tumor progression and enhanced cell death, resulting in high levels of nucleosomes entering into the bloodstream [50, 52]. In addition, cancer patients with a large number of necrotic tumor cells in advanced stage have more plasma ctDNA than the patients in the early stage [10]. These data support the view that apoptotic or necrotic tumor cells are likely to be the main origin of ctDNA.

However, cancer patients in the early stage also contain plasma ctDNA [2, 10], and therefore it is likely that apoptotic or necrotic tumor cells are not the only source. *In vitro* studies found that living tumor cells, like lymphocytes, can continuously and automatically release DNA [49], which might explain the presence of detectable ctDNA in patients with early-stage cancer. In addition, the amount of ctDNA increases with tumor growth [13], further supporting the hypothesis that ctDNA might be derived from living tumor cells. Evidence also supports a third scenario, in which DNA is released from CTC. Firstly, ctDNA and CTC have been shown to contain identical genetic mutations. Also, CTC can evade the macrophage clearance and easily enter the blood. Moreover, it has also been suggested that blood that contains CTC also contains ctDNA [10]. These findings support the view that CTC might be another source of ctDNA. Since peripheral blood only contains a few of CTC, ctDNA from CTC might not be the main origin.

### The biological function of ctDNA

Generally, metastases represent the end-products of the invasion-metastasis cascade, which involves the development of the invasiveness capacity of cells in primary tumors, with subsequent blood dissemination of such cells and extravasation and metastasis to distant sites [53, 54]. However, this theory has been challenged in the last decades owing to the fact that accumulating evidence has indicated ctDNA might play a key role in cancer metastasis through oncogenic transformation of susceptible cells [54–57].

In 1999, García-Olmo et al. showed that plasma from tumor bearing rats could stably transform normal cells cultured *in vitro* and for the first time proposed the hypothesis of genometastasis: “metastasis might occur *via* transfection of susceptible cells, located in distant target organs, with dominant oncogenes that are derived from the primary tumor and are circulating in the plasma” [55]. Consistently, the serum of cancer patients and supernatant of human cancer cells were also able to induce *in vitro* normal cell transformation and tumorigenesis, while this process did not occur if serum and supernatants were deprived of DNA [57, 58]. In subsequent studies, García-Olmo et al. found in untreated, tumor-bearing rats as well as in surgically treated ones that hematogenous dissemination of tumors appeared to be more closely related to ctDNA than to CTC [59, 60]. It was also showed in rats that not only the infection of tumor cells but also the recruitment of host cells was essential for tumor formation [61]. Furthermore, Roth C et al. found the presence of distant metastasis associated with a significant increase in DNA levels [50]. Their findings also supported the previous observations of Diehl F et al., which suggested that as tumors invaded through the intestine to distant sites, the number of ctDNA molecules progressively increased [27]. Intriguingly, ctDNA could be horizontally transferred between the tumor cells and normal cells *via* uptake of apoptotic bodies or virtosomes [54, 58, 62], resulting in distant metastasis (Figure 1). Taken together, these data showed that ctDNA might have properties for integrating into susceptible cell genome and transforming these cells oncogenically.

The hypothesis of Genometastasis seems to be a reasonable explanation for cancer metastasis. However, present studies were still not sufficiently potent to prove the pro-metastasis function of ctDNA in consideration of incomplete study design and the absence of trails *in vivo*, especially in clinic. It is necessary to further verify in the future.

## METHODOLOGIES FOR DETECTION OF ctDNA

Detection of plasma ctDNA is not only good for the study of cancer pathogenesis, but also beneficial to the clinical management of cancer. Because ctDNA has been shown to possess the characteristic mutations of the corresponding primary tumor, researchers have tried to take advantage of this feature in designing assays that may be used in cancer management. However, ctDNA-based assays are confronted with several challenges, not least that ctDNA accounts for only a small percentage (sometimes < 0.01%) of the total cell-free DNA in the peripheral blood and that prior knowledge about particular mutations is usually required, which may be hard to obtain [2].

Originally, researchers used Sanger sequencing to detect plasma ctDNA. However, there are many shortcomings for Sanger-based ctDNA detection, such as low-throughput, laborious protocols, high cost, and potential bias introduced by the PCR methodology [13]. In the last decade, the advances in NGS technology have allowed researchers to develop many effective and convenient alternatives to Sanger sequencing. Diehl et al. developed a technique called BEAMing (beads, emulsion, amplification, and magnetics) to detect ctDNA in blood [20]. In this technique, the object DNA segment is amplified using primers containing known tag sequences, and then covalently bound to magnetic beads. Finally, flow cytometry is used to sort beads containing the mutation. Newman et al. developed another new technique called CAPP-seq (cancer personalized profiling by deep sequencing) for quantifying ctDNA. They designed a probe panel consisting of biotinylated DNA oligonucleotides that target recurrently mutated regions in the cancer of interest. Using this technique, they detected ctDNA in 100% of stage II-IV and 50% of stage I NSCLC patients, with 96% specificity for mutant allele fractions down to ~0.02% [13]. Compared with previous methods, these new techniques provide significantly higher sensitivity for ctDNA detection. They are also high-throughput and less expensive. Such “second generation” sequencing techniques have been essential in fully evaluating the clinical potential of ctDNA analysis. However, there are also limitations to these novel techniques. Firstly, NGS-based methods provide an informative diagnosis in only around 50% of early-stage patients [2, 13], therefore the sensitivity requires further improvement. Additionally, the costs remain relatively high, limiting their application in clinical practice. Fortunately, third-generation sequencing techniques, designed to be highly sensitive and inexpensive, have rapidly advanced and have the potential to expedite extensive application of ctDNA detection for routine patient management [63–69].

## THE CRITICAL ROLE OF ctDNA IN CANCER DIAGNOSIS AND PROGNOSIS

Tissue biopsy is still the gold standard for tumor diagnosis. However, many shortcomings exist. For instance, there are risks associated with invasive sampling, particularly when applied to the fragile organs such as lung, and sensitivity is suboptimal, frequently resulting in an inability to detect early-stage tumors. In addition, because tumors are heterogeneous and constantly evolving [70–75], tissue biopsy-based investigations are often unable to accurately determine tumor progression [2]. Similarly, they also struggle to detect small, residual lesions following therapy. In recent years, it has been suggested that a plasma biomarker-based approach can evaluate the tumor occurrence, progression and recurrence. Such approach is minimally invasive with satisfactory conformity [3]. However, previous tumor biomarkers in plasma only provide limited sensitivity and specificity, and therefore cannot always meet the clinical requirements [2, 10].

ctDNA has now been widely evaluated as a novel biomarker for liquid biopsy in cancer diagnosis and prognosis [2, 3]. Liquid biopsy based on ctDNA is superior to that of previous plasma biomarkers in two main areas: 1) sensitivity, and 2) clinical correlations. AFP, CEA, PSA, and CA15-3 are plasma protein biomarkers commonly used in clinical management [76–81]. However, the positive proportion of patients for these biomarkers is usually between 50~70% in cancer patients. Furthermore, they are also found in the serum of individuals without cancer, although in lower concentrations [2, 10, 82, 83]. Dawson et al. assessed ctDNA, CTC, and CA15-3 in 30 metastatic breast cancer patients and found that the detection rate of ctDNA reached 97%, whereas rates for CTC and CA15-3 were only 78% and 87%, respectively [78]. Bettegowda et al., in a study of 206 metastatic cancer patients, found that the sensitivity of ctDNA detection was 87.2% [10]. In their further studies, ctDNA and CTC were tested in 16 cancer patients, revealing that 13 patients who were ctDNA-positive were negative by CTC test. The three patients with both ctDNA and CTC positive contained 50-fold more ctDNA than DNA obtained from CTC [10]. Together, these studies indicate that ctDNA is more sensitive than protein biomarkers and CTC.

Importantly, the half-life of ctDNA is less than 2 hours, whereas the half-life of protein markers in plasma can be several weeks. This means that ctDNA can more accurately reflect the real-time tumor burden in patients receiving therapy. Indeed, ctDNA has been shown to correlate well with tumor load and likelihood of recurrence. Researchers tested blood samples from various cancer patients and found that ctDNA were present at significantly different levels among the patients with different cancer stages. Specifically, patients with advanced-stages of gastro-esophageal, pancreatic, breast and colorectal cancer had a higher level of ctDNA than patients in early stages of those diseases [10, 84]. Additionally, researchers analyzed ctDNA in relapsing and non-relapsing patients, and found that ctDNA could be used to monitor relapse status, resulting a 10-month lead-time on detection of relapse compared with the conventional follow-up [35]. A study by Garcia-Murillas I et al. found similar results [85]. Therefore, ctDNA has potential to be used for the evaluation of tumor progression and prognostic. In summary, liquid biopsy based on ctDNA analysis might represent the next generation of tumor diagnostic and prognostic testing on account of its high accuracy and sensitivity.

## THE CRITICAL ROLE OF ctDNA IN CANCER TREATMENT

### Genotyping and assistance in personalized, targeted therapy

Genotyping is aimed at the analysis of genetic mutations and has important applications in managing cancer treatment [86–89]. In recent years, it has been suggested that genotyping has the potential to play a crucial role in precision medicine, especially in precision immunotherapy, *via* facilitating the development of new therapeutic protocols based on known or unknown key genetic lesions [90]. For example, in the exploitation of precision immunotherapy strategies, researchers can potentially screen for the antigens that induce strong immune responses according to the genotyping information and then find and enrich the T cells that can be used in personalized therapy to target those new antigens [90].

In current clinical practice, genotyping is achieved using DNA obtained from the tissue biopsy. However, tissue biopsy can only obtain local and static tumor information, and is unable to reflect the real-time tumor genotyping due to heterogeneity and constant evolution of tumors [1]. ctDNA analysis overcomes these problems by reflecting the genetic mutations of the whole tumor tissue. Additionally, ctDNA from the same patients at different stages can be used to dynamically monitor the genetic mutations during the cancer progression [14, 91]. Therefore, Liquid biopsy based on ctDNA analysis might improve tumor genotyping and targeted cancer therapy, which would be of significant benefit to the field of personalized medicine.

### Disease monitoring and treatment evaluation

Cancer often relapses and evolves during the treatment. Disease monitoring and treatment evaluation are important for clinician to determine subsequent treatment protocols. It has been shown that serial ctDNA detection can be used to evaluate treatment efficacy by assessing remission status and detecting relapse and progression [10, 20, 92–95]. Using ctDNA, the detection rates among the patients with stage I, II, III, and IV cancer were 47, 55, 69, and 82%, indicating that ctDNA levels increase with cancer progression [10]. Additionally, Diehl et al. found that majority of the patients had significantly decreased or absent ctDNA levels after surgery. Further follow-up studies suggested that the patients with detectable ctDNA after surgery all relapsed, while those without detectable ctDNA after surgery remained in remission [20]. Reinert et al. found that there was 10 months' lead-time on detection of relapse using ctDNA detection compared with conventional follow-up [92]. These studies indicate that ctDNA detection can quickly predict the therapy outcomes, and provide an important reference for clinicians in determining the next treatment protocols.

### Illustration of the mechanisms of therapy resistance

Therapy resistance is the major cause of cancer treatment failure. However, so far appropriate methods of investigating tumor resistance have not yet been established [37, 96]. Recently, studies on ctDNA have provided new insight into this area [96–101]. Diaz et al. analyzed the ctDNA of lung cancer patients who were subject to EGFR inhibitor treatment and found 42 *KRAS* gene mutations, which are known to be markers of therapy resistance. Further studies showed that this method could provide 5 months' lead-time on detection of tumor evolution and resistance compared with the conventional method [97, 98]. In addition, Murtaza M et al. collected blood samples from six patients with advanced-stage breast cancer, lung cancer or ovarian cancer, and performed whole-exome sequencing. They found frequent mutations in genes involved in pathways relevant to the development of cancer resistance. For instance, they found the mutations that blocked the binding of drug to its biological targets [96]. Therefore, ctDNA detection can be used to monitor cancer evolution, illustrate the mechanisms of the tumor resistance, and guide drug selection for the clinicians.

## IMPLICATIONS AND FUTURE DIRECTIONS

Recent advances in our understanding of the biology and clinical application of ctDNA have provided evidence that the use of ctDNA as a liquid biopsy can improve cancer diagnosis and treatment *via* genotyping, disease monitoring, treatment evaluation, and so on. However, there are still some challenges that should be addressed so that this technique may eventually be implemented into routine clinical practice. Firstly, to use ctDNA as a diagnostic marker, it will be important to gain a better understanding of the biological characteristics of ctDNA, including its size, existing form, and the mechanisms by which ctDNA is released into the bloodstream. Additionally, though the clinical correlations of ctDNA analysis have been verified, adoption of this technique into routine clinical practice still need to further demonstrate its analytic validity, clinical validity, and, most importantly, clinical utility in consideration of the suboptimal sensitivity of the methods for ctDNA detection and the limited sample quantity. These challenges indicate that it will take some time to introduce ctDNA analysis into clinical practice [2, 102]. However, as sequencing technologies quickly develop, and the understanding of ctDNA biology and clinical potential deepens, the eventual use of ctDNA in clinical practice seems to be assured.

## References

[1] Gerlinger M, Rowan AJ, Horswell S, Larkin J, Endesfelder D, Gronroos E, Martinez P, Matthews N, Stewart A, Tarpey P, Varela I, Phillimore B, Begum S (2012). Intratumor heterogeneity and branched evolution revealed by multiregion sequencing. N Engl J Med.

[2] Yong E (2014). Cancer biomarkers: Written in blood. Nature.

[3] Mäbert K, Cojoc M, Peitzsch C, Kurth I, Souchelnytskyi S, Dubrovska A (2014). Cancer biomarker discovery: current status and future perspectives. Int J Radiat Biol.

[4] Ballehaninna UK, Chamberlain RS (2011). Serum CA19-9 as a biomarker for pancreatic cancer—A comprehensive review. Indian J Surg Oncol.

[5] Han SX, Wang JL, Guo XJ, He CC, Ying X, Ma JL, Zhang YY, Zhao Q, Zhu Q (2014). Serum SALL4 is a novel prognosis biomarker with tumor recurrence and poor survival of patients in hepatocellular carcinoma. J Immunol Res.

[6] Han SX, Zhou X, Sui X, He CC, Cai MJ, Ma JL, Zhang YY, Zhou CY, Ma CX, Varela-Ramirez A, Zhu Q (2015). Serum dickkopf-1 is a novel serological biomarker for the diagnosis and prognosis of pancreatic cancer. Oncotarget.

[7] Kosaka N, Iguchi H, Ochiya T (2010). Circulating microRNA in body fluid: a new potential biomarker for cancer diagnosis and prognosis. Cancer Sci.

[8] Shen J, Hu Q, Schrauder M, Yan L, Wang D, Medico L, Guo Y, Yao S, Zhu Q, Liu B, Qin M, Beckmann MW, Fasching PA (2014). Circulating miR-148b and miR-133a as biomarkers for breast cancer detection. Oncotarget.

[9] Yap TA, Lorente D, Omlin A, Olmos D, de Bono JS (2014). Circulating tumor cells: a multifunctional biomarker. Clin Cancer Res.

[10] Bettegowda C, Sausen M, Leary RJ, Kinde I, Wang Y, Agrawal N, Bartlett BR, Wang H, Luber B, Alani RM, Antonarakis ES, Azad NS, Bardelli A (2014). Detection of circulating tumor DNA in early- and late-stage human malignancies. Sci Transl Med.

[11] Diamandis EP (2012). The failure of protein cancer biomarkers to reach the clinic: why and what can be done to address the problem?. BMC Med.

[12] Sorenson GD, Pribish DM, Valone FH, Memoli VA, Bzik DJ, Yao SL (1994). Soluble normal and mutated DNA sequences from single-copy genes in human blood. Cancer Epidemiol Biomarkers Prev.

[13] Newman AM, Bratman SV, To J, Wynne JF, Eclov NC, Modlin LA, Liu CL, Neal JW, Wakelee HA, Merritt RE, Shrager JB, Loo BW, Alizadeh AA (2014). An ultrasensitive method for quantitating circulating tumor DNA with broad patient coverage. Nat Med.

[14] Diaz LA, Bardelli A (2014). Liquid biopsies: genotyping circulating tumor DNA. J Clin Oncol.

[15] Romero D (2015). Breast cancer: Tracking ctDNA to evaluate relapse risk. Nat Rev Clin Oncol.

[16] Bidard FC, Madic J, Mariani P, Piperno-Neumann S, Rampanou A, Servois V, Cassoux N, Desjardins L, Milder M, Vaucher I, Pierga JY, Lebofsky R, Stern MH (2014). Detection rate and prognostic value of circulating tumor cells and circulating tumor DNA in metastatic uveal melanoma. Int J Cancer.

[17] Martignetti JA, Camacho-Vanegas O, Priedigkeit N, Camacho C, Pereira E, Lin L, Garnar-Wortzel L, Miller D, Losic B, Shah H, Liao J, Ma J, Lahiri P (2014). Personalized ovarian cancer disease surveillance and detection of candidate therapeutic drug target in circulating tumor DNA. Neoplasia.

[18] Leon SA, Shapiro B, Sklaroff DM, Yaros MJ (1977). Free DNA in the serum of cancer patients and the effect of therapy. Cancer Res.

[19] Vasioukhin V, Anker P, Maurice P, Lyautey J, Lederrey C, Stroun M (1994). Point mutations of the N-ras gene in the blood plasma DNA of patients with myelodysplastic syndrome or acute myelogenous leukaemia. Br J Haematol.

[20] Diehl F, Schmidt K, Choti MA, Romans K, Goodman S, Li M, Thornton K, Agrawal N, Sokoll L, Szabo SA, Kinzler KW, Vogelstein B, Diaz LA (2008). Circulating mutant DNA to assess tumor dynamics. Nat Med.

[21] Kruglyak KM, Lin E, Ong FS (2014). Next-generation sequencing in precision oncology: challenges and opportunities. Expert Rev Mol Diagn.

[22] Lebofsky R, Decraene C, Bernard V, Kamal M, Blin A, Leroy Q, Rio Frio T, Pierron G, Callens C, Bieche I, Saliou A, Madic J, Rouleau E (2015). Circulating tumor DNA as a non-invasive substitute to metastasis biopsy for tumor genotyping and personalized medicine in a prospective trial across all tumor types. Mol Oncol.

[23] Chaudhuri AA, Binkley MS, Osmundson EC, Alizadeh AA, Diehn M (2015). Predicting Radiotherapy Responses and Treatment Outcomes Through Analysis of Circulating Tumor DNA. Semin Radiat Oncol.

[24] Nie K, Jia Y, Zhang X (2015). Cell-free circulating tumor DNA in plasma/serum of non-small cell lung cancer. Tumour Biol.

[25] Chan KC, Jiang P, Zheng YW, Liao GJ, Sun H, Wong J, Siu SS, Chan WC, Chan SL, Chan AT, Lai PB, Chiu RW, Lo YM (2013). Cancer genome scanning in plasma: Detection of tumor-associated copy number aberrations single-nucleotide variants and tumoral heterogeneity by massively parallel sequencing. Clin Chem.

[26] Yung TK, Chan KC, Mok TS, Tong J, To KF, Lo YM (2009). Single-molecule detection of epidermal growth factor receptor mutations in plasma by microfluidics digital PCR in non-small cell lung cancer patients. Clin Cancer Res.

[27] Diehl F, Li M, Dressman D, He Y, Shen D, Szabo S, Diaz LA, Goodman SN, David KA, Juhl H, Kinzler KW, Vogelstein B (2005). Detection and quantification of mutations in the plasma of patients with colorectal tumors. Proc Natl Acad Sci USA.

[28] Qiu M, Wang J, Xu Y, Ding X, Li M, Jiang F, Xu L, Yin R (2015). Circulating tumor DNA is effective for the detection of EGFR mutation in non-small cell lung cancer: a meta-analysis. Cancer Epidemiol Biomarkers Prev.

[29] Freidin MB, Freydina DV, Leung M, Montero Fernandez A, Nicholson AG, Lim E (2015). Circulating Tumor DNA Outperforms Circulating Tumor Cells for KRAS Mutation Detection in Thoracic Malignancies. Clin Chem.

[30] Wong IH, Lo YM, Zhang J, Liew CT, Ng MH, Wong N, Lai PB, Lau WY, Hjelm NM, Johnson PJ (1999). Detection of aberrant p16 methylation in the plasma and serum of liver cancer patients. Cancer Res.

[31] Chan KC, Lai PB, Mok TS, Chan HL, Ding C, Yeung SW, Lo YM (2008). Quantitative analysis of circulating methylated DNA as a biomarker for hepatocellular carcinoma. Clin Chem.

[32] Chan KC, Jiang P, Chan CW, Sun K, Wong J, Hui EP, Chan SL, Chan WC, Hui DS, Ng SS, Chan HL, Wong CS, Ma BB (2013). Noninvasive detection of cancer-associated genome-wide hypomethylation and copy number aberrations by plasma DNA bisulfite sequencing. Proc Natl Acad Sci USA.

[33] Balgkouranidou I, Chimonidou M, Milaki G, Tsarouxa EG, Kakolyris S, Welch DR, Georgoulias V, Lianidou ES (2014). Breast cancer metastasis suppressor-1 promoter methylation in cell-free DNA provides prognostic information in non-small cell lung cancer. Br J Cancer.

[34] Lo YM, Chan LY, Lo KW, Leung SF, Zhang J, Chan AT, Lee JC, Hjelm NM, Johnson PJ, Huang DP (1999). Quantitative analysis of cell-free Epstein-Barr virus DNA in plasma of patients with nasopharyngeal carcinoma. Cancer Res.

[35] Chan KC, Hung EC, Woo JK, Chan PK, Leung SF, Lai FP, Cheng AS, Yeung SW, Chan YW, Tsui TK, Kwok JS, King AD, Chan AT (2013). Early detection of nasopharyngeal carcinoma by plasma Epstein-Barr virus DNA analysis in a surveillance program. Cancer.

[36] Campitelli M, Jeannot E, Peter M, Lappartient E, Saada S, de la Rochefordière A, Fourchotte V, Alran S, Petrow P, Cottu P, Pierga JY, Lantz O, Couturier J (2012). Human papillomavirus mutational insertion: specific marker of circulating tumor DNA in cervical cancer patients. PLoS One.

[37] Forshew T, Murtaza M, Parkinson C, Gale D, Tsui DW, Kaper F, Dawson SJ, Piskorz AM, Jimenez-Linan M, Bentley D, Hadfield J, May AP, Caldas C (2012). Noninvasive identification and monitoring of cancer mutations by targeted deep sequencing of plasma DNA. Sci Transl Med.

[38] Chang-HaoTsao S, Weiss J, Hudson C, Christophi C, Cebon J, Behren A, Dobrovic A (2015). Monitoring response to therapy in melanoma by quantifying circulating tumor DNA with droplet digital PCR for BRAF and NRAS mutations. Sci Rep.

[39] Thierry AR, Mouliere F, Gongora C, Ollier J, Robert B, Ychou M, Del Rio M, Molina F (2010). Origin and quantification of circulating DNA in mice with human colorectal cancer xenografts. Nucleic Acids Res.

[40] Kidess E, Heirich K, Wiggin M, Vysotskaia V, Visser BC, Marziali A, Wiedenmann B, Norton JA, Lee M, Jeffrey SS, Poultsides GA (2015). Mutation profiling of tumor DNA from plasma and tumor tissue of colorectal cancer patients with a novel high-sensitivity multiplexed mutation detection platform. Oncotarget.

[41] Wang BG, Huang HY, Chen YC, Bristow RE, Kassauei K, Cheng CC, Roden R, Sokoll LJ, Chan DW, Shih IeM (2003). Increased plasma DNA integrity in cancer patients. Cancer Res.

[42] Gao YJ, He YJ, Yang ZL, Shao HY, Zuo Y, Bai Y, Chen H, Chen XC, Qin FX, Tan S, Wang J, Wang L, Zhang L (2010). Increased integrity of circulating cell-free DNA in plasma of patients with acute leukemia. Clin Chem Lab Med.

[43] Mouliere F, Robert B, ArnauPeyrotte E, Del Rio M, Ychou M, Molina F, Gongora C, Thierry AR (2011). High fragmentation characterizes tumour-derived circulating DNA. PLoS One.

[44] Jiang P, Chan CW, Chan KC, Cheng SH, Wong J, Wong VW, Wong GL, Chan SL, Mok TS, Chan HL, Lai PB, Chiu RW, Lo YM (2015). Lengthening and shortening of plasma DNA in hepatocellular carcinoma patients. Proc Natl Acad Sci USA.

[45] Madhavan D, Wallwiener M, Bents K, Zucknick M, Nees J, Schott S, Cuk K, Riethdorf S, Trumpp A, Pantel K, Sohn C, Schneeweiss A, Surowy H (2014). Plasma DNA integrity as a biomarker for primary and metastatic breast cancer and potential marker for early diagnosis. Breast Cancer Res Treat.

[46] Mouliere F, Rosenfeld N (2015). Circulating tumor-derived DNA is shorter than somatic DNA in plasma. Proc Natl Acad Sci USA.

[47] Anker P, Mulcahy H, Chen XQ, Stroun M (1999). Detection of circulating tumour DNA in the blood (plasma/serum) of cancer patients. Cancer Metastasis Rev.

[48] Stroun M, Lyautey J, Lederrey C, Olson-Sand A, Anker P (2001). About the possible origin and mechanism of circulating DNA: apoptosis and active DNA release. Clin Chim Acta.

[49] van der Vaart M, Pvetorius PJ (2007). The origin of circulating free DNA. Clin Chem.

[50] Roth C, Pantel K, Müller V, Rack B, Kasimir-Bauer S, Janni W, Schwarzenbach H (2011). Apoptosis-related deregulation of proteolytic activities and high serum levels of circulating nucleosomes and DNA in blood correlate with breast cancer progression. BMC Cancer.

[51] López-Otín C, Matrisian LM (2007). Emerging roles of proteases in tumour suppression. Nat Rev Cancer.

[52] Holdenrieder S, Nagel D, Schalhorn A, Heinemann V, Wilkowski R, von Pawel J, Raith H, Feldmann K, Kremer AE, Müller S, Geiger S, Hamann GF, Seidel D (2008). Clinical relevance of circulating nucleosomes in cancer. Ann N Y Acad Sci.

[53] Valastyan S, Weinberg RA (2011). Tumor Metastasis: molecular insights and evolving paradigms. Cell.

[54] García-Olmo DC, García-Olmo D (2013). Biological Role of Cell-Free Nucleic Acids in Cancer: The Theory of Genometastasis. Crit Rev Oncog.

[55] García-Olmo D, García-Olmo DC, Ontañón J, Martinez E, Vallejo M (1999). Tumor DNA circulating in the plasma might play a role in metastasis The hypothesis of the genometastasis. Histol Histopathol.

[56] García-Olmo D, García-Olmo DC (2001). Functionality of circulating DNA: the hypothesis of genometastasis. Ann N Y Acad Sci.

[57] García-Olmo DC, Domínguez C, García-Arranz M, Anker P, Stroun M, García-Verdugo JM, García-Olmo D (2010). Cell-free nucleic acids circulating in the plasma of colorectal cancer patients induce the oncogenic transformation of susceptible cultured cells. Cancer Res.

[58] Trejo-Becerril C, Pérez-Cárdenas E, Taja-Chayeb L, Anker P, Herrera-Goepfert R, Medina-Velázquez LA, Hidalgo-Miranda A, Pérez-Montiel D, Chávez-Blanco A, Cruz-Velázquez J, Díaz-Chávez J, Gaxiola M, Dueñas-González A (2012). Cancer progression mediated by horizontal gene transfer in an *in vivo* model. PLoS One.

[59] García-Olmo DC, Gutiérrez-González L, Ruiz-Piqueras R, Picazo MG, García-Olmo D (2005). Detection of circulating tumor cells and of tumor DNA in plasma during tumor progression in rats. Cancer Lett.

[60] García-Olmo DC, Gutiérrez-González L, Samos J, Picazo MG, Atiénzar M, García-Olmo D (2006). Surgery and hematogenous dissemination: comparison between the detection of circulating tumor cells and of tumor DNA in plasma before and after tumor resectioninrats. Ann Surg Oncol.

[61] García-Olmo DC, Samos J, Picazo MG, Fernández-Miguel G, Toboso I, García-Olmo D (2008). Loss of a reporter gene for grDiehl Feen fluorescent protein during tumor progression suggests the recruitment of host cells in rats with experimentally induced colon cancer. Histol Histopathol.

[62] Gahan PB, Stroun M (2010). The virtosome-a novel cytosolic informative entity and intercellular messenger. Cell Biochem Funct.

[63] Laszlo AH, Derrington IM, Ross BC, Brinkerhoff H, Adey A, Nova IC, Craig JM, Langford KW, Samson JM, Daza R, Doering K, Shendure J, Gundlach JH (2014). Decoding long nanopore sequencing reads of natural DNA. Nat Biotechnol.

[64] Rosenstein J (2014). The promise of nanopore technology: Nanopore DNA sequencing represents a fundamental change in the way that genomic information is read with potentially big savings. IEEE Pulse.

[65] Steinbock LJ, Radenovic A (2015). The emergence of nanopores in next-generation sequencing. Nanotechnology.

[66] Atas E, Singer A, Meller A (2012). DNA sequencing and bar-coding using solid-state nanopores. Electrophoresis.

[67] Bayley H (2015). Nanopore sequencing: from imagination to reality. Clin Chem.

[68] Derrington IM, Butler TZ, Collins MD, Manrao E, Pavlenok M, Niederweis M, Gundlach JH (2010). Nanopore DNA sequencing with MspA. Proc Natl Acad Sci USA.

[69] Smith CC, Wang Q, Chin CS, Salerno S, Damon LE, Levis MJ, Perl AE, Travers KJ, Wang S, Hunt JP, Zarrinkar PP, Schadt EE, Kasarskis A (2012). Validation of ITD mutations in FLT3 as a therapeutic target in human acute myeloid leukaemia. Nature.

[70] Parsons DW, Jones S, Zhang X, Lin JC, Leary RJ, Angenendt P, Mankoo P, Carter H, Siu IM, Gallia GL, Olivi A, McLendon R, Rasheed BA (2008). An integrated genomic analysis of human glioblastoma multiforme. Science.

[71] Mardis ER, Ding L, Dooling DJ, Larson DE, McLellan MD, Chen K, Koboldt DC, Fulton RS, Delehaunty KD, McGrath SD, Fulton LA, Locke DP, Magrini VJ (2009). Recurring mutations found by sequencing an acute myeloid leukemia genome. N Engl J Med.

[72] Campbell PJ, Yachida S, Mudie LJ, Stephens PJ, Pleasance ED, Stebbings LA, Morsberger LA, Latimer C, McLaren S, Lin ML, McBride DJ, Varela I, Nik-Zainal (2010). The patterns and dynamics of genomic instability in metastatic pancreatic cancer. Nature.

[73] Navin N, Kendall J, Troge J, Andrews P, Rodgers L, McIndoo J, Cook K, Stepansky A, Levy D, Esposito D, Muthuswamy L, Krasnitz A, McCombie WR (2011). Tumour evolution inferred by single-cell sequencing. Nature.

[74] Boesch M, Zeimet AG, Reimer D, Schmidt S, Gastl G, Parson W, Spoeck F, Hatina J, Wolf D, Sopper S (2014). The side population of ovarian cancer cells defines a heterogeneous compartment exhibiting stem cell characteristics. Oncotarget.

[75] Hewer E, Beck J, Murek M, Kappeler A, Vassella E, Vajtai I (2014). Polymorphous oligodendroglioma of Zülch revisited: a genetically heterogeneous group of anaplastic gliomas including tumors of bona fide oligodendroglial differentiation. Neuropathology.

[76] Tachibana M, Takemoto Y, Nakashima Y, Kinugasa S, Kotoh T, Dhar DK, Kohno H, Nagasue N (1998). Serum carcinoembryonic antigen as a prognostic factor in resectable gastric cancer. J Am Coll Surg.

[77] Ahrens MJ, Bertin PA, Vonesh EF, Meade TJ, Catalona WJ, Georganopoulou D (2013). PSA enzymatic activity: a new biomarker for assessing prostate cancer aggressiveness. Prostate.

[78] Dawson SJ, Tsui DW, Murtaza M, Biggs H, Rueda OM, Chin SF, Dunning MJ, Gale D, Forshew T, Mahler-Araujo B, Rajan S, Humphray S, Becq J (2013). Analysis of circulating tumor DNA to monitor metastatic breast cancer. N Engl J Med.

[79] He CZ, Zhang KH, Li Q, Liu XH, Hong Y, Lv NH (2013). Combined use of AFP CEA CA125 and CAl9-9 improves the sensitivity for the diagnosis of gastric cancer. BMC Gastroenterol.

[80] Duffy MJ (2001). Screening for prostate cancer: Carcinoembryonic antigen as a marker for colorectal cancer: is it clinically useful?. Clin Chem.

[81] Mazzucchelli R, Colanzi P, Pomante R, Muzzonigro G, Montironi R (2000). Prostate tissue and serum markers. Adv Clin Pathol.

[82] RuibalMorell A (1992). CEA serum levels in non-neoplastic disease. Int J Biol Markers.

[83] Gomella LG, Liu XS, Trabulsi EJ, Kelly WK, Myers R, Showalter T, Dicker A, Wender R (2011). Screening for prostate cancer: the current evidence and guidelines controversy. Can J Urol.

[84] Fleischhacker M, Schmidt B (2007). Circulating nucleic acids (CNAs) and cancer—a survey. Biochim Biophys Acta.

[85] Garcia-Murillas I, Schiavon G, Weigelt B, Ng C, Hrebien S, Cutts RJ, Cheang M, Osin P, Nerurkar A, Kozarewa I, Garrido JA, Dowsett M, Reis-Filho JS (2015). Mutation tracking in circulating tumor DNA predicts relapse in early breast cancer. Sci Transl Med.

[86] Li T, Kung HJ, Mack PC, Gandara DR (2013). Genotyping and genomic profiling of non-small-cell lung cancer: implications for current and future therapies. J Clin Oncol.

[87] Damkier P (2014). CYP2D6 genotyping and tamoxifen in the treatment of post-menopausal breast cancer. Br J Clin Pharmacol.

[88] Huh WK, Williams E, Huang J, Bramley T, Poulios N (2015). Cost effectiveness of human papillomavirus-16/18 genotyping in cervical cancer screening. Appl Health Econ Health Policy.

[89] Stachler MD, Rinehart E, Lindeman N, Odze R, Srivastava A (2015). Novel molecular insights from routine genotyping of colorectal carcinomas. Hum Pathol.

[90] Kreiter S, Vormehr M, van de Roemer N, Diken M, Löwer M, Diekmann J, Boegel S, Schrörs B, Vascotto F, Castle JC, Tadmor AD, Schoenberger SP, Huber C (2015). Mutant MHC class II epitopes drive therapeutic immune responses to cancer. Nature.

[91] Hamakawa T, Kukita Y, Kurokawa Y, Miyazaki Y, Takahashi T, Yamasaki M, Miyata H, Nakajima K, Taniguchi K, Takiguchi S, Mori M, Doki Y, Kato K (2015). Monitoring gastric cancer progression with circulating tumour DNA. Br J Cancer.

[92] Reinert T, Schøler LV, Thomsen R, Tobiasen H, Vang S, Nordentoft I, Lamy P, Kannerup AS, Mortensen FV, Stribolt K, Hamilton-Dutoit S, Nielsen HJ, Laurberg S (2015). Analysis of circulating tumor DNA to monitor disease burden following colorectal cancer surgery. Gut.

[93] Lipson EJ, Velculescu VE, Pritchard TS, Sausen M, Pardoll DM, Topalian SL, Diaz LA (2014). Circulating tumor DNA analysis as a real-time method for monitoring tumor burden in melanoma patients undergoing treatment with immune checkpoint blockade. J Immunother Cancer.

[94] Roschewski M, Dunleavy K, Pittaluga S, Moorhead M, Pepin F, Kong K, Shovlin M, Jaffe ES, Staudt LM, Lai C, Steinberg SM, Chen CC, Zheng J (2015). Circulating tumor DNA and CT monitoring in patients with untreated diffuse large B-cell lymphoma: a correlative biomarker study. Lancet Oncol.

[95] Tie J, Kinde I, Wang Y, Wong HL, Roebert J, Christie M, Tacey M, Wong R, Singh M, Karapetis CS, Desai J, Tran B, Strausberg RL (2015). Circulating tumor DNA as an early marker of therapeutic response in patients with metastatic colorectal cancer. Ann Oncol.

[96] Murtaza M, Dawson SJ, Tsui DW, Gale D, Forshew T, Piskorz AM, Parkinson C, Chin SF, Kingsbury Z, Wong AS, Marass F, Humphray S, Hadfield J (2013). Non-invasive analysis of acquired resistance to cancer therapy by sequencing of plasma DNA. Nature.

[97] Diaz LA, Williams RT, Wu J, Kinde I, Hecht, Berlin J, Allen B, Bozic I, Reiter JG, Nowak MA, Kinzler KW, Oliner KS, Vogelstein B (2012). The molecular evolution of acquired resistance to targeted EGFR blockade in colorectal cancers. Nature.

[98] Taniguchi K, Uchida J, Nishino K, Kumagai T, Okuyama T, Okami J, Higashiyama M, Kodama K, Imamura F, Kato K (2011). Quantitative detection of EGFR mutations in circulating tumor DNA derived from lung adenocarcinomas. Clin Cancer Res.

[99] Siravegna G, Mussolin B, Buscarino M, Corti G, Cassingena A, Crisafulli G, Ponzetti A, Cremolini C, Amatu A, Lauricella C, Lamba S, Hobor S, Avallone A (2015). Clonal evolution and resistance to EGFR blockade in the blood of colorectal cancer patients. Nat Med.

[100] Gray ES, Rizos H, Reid AL, Boyd SC, Pereira MR, Lo J, Tembe V, Freeman J, Lee JH, Scolyer RA, Siew K, Lomma C, Cooper A (2015). Circulating tumor DNA to monitor treatment response and detect acquired resistance in patients with metastatic melanoma. Oncotarget.

[101] Jiang T, Ren S, Zhou C (2015). Role of circulating-tumor DNA analysis in non-small cell lung cancer. Lung Cancer.

[102] Ilie M, Hofman V, Long E, Bordone O, Selva E, Washetine K, Marquette CH, Hofman P (2014). Current challenges for detection of circulating tumor cells and cell-free circulating nucleic acids, and their characterization in non-small cell lung carcinoma patients. What is the best blood substrate for personalized medicine?. Ann Transl Med.

